# Causal association between varicose veins and atrial fibrillation: A 2-sample bidirectional Mendelian randomization study

**DOI:** 10.1097/MD.0000000000041466

**Published:** 2025-02-14

**Authors:** Weiyue Chen, Na Jing, Qingzhi Liu, Hong Mao, Xiangyu Wang, Boxun Chen, Yannan Wang

**Affiliations:** a First Clinical College, Shandong University of Traditional Chinese Medicine, Jinan, China; b Department of Vascular Surgery, Affiliated Hospital of Shandong University of Traditional Chinese Medicine, Jinan, China.

**Keywords:** atrial fibrillation, GWAS, Mendelian randomization, varicose veins

## Abstract

This study selected genome-wide association study data from the FinnGen database and utilized a bidirectional 2-sample Mendelian randomization (MR) method to explore the causal association between varicose veins (VV) and atrial fibrillation (AF). Inverse variance weighted (IVW) was used as the primary analytical method to assess the causal relationship between VV and AF, supplemented by Weighted median, MR-Egger and Simple 1mode. Cochran’s *Q* test, MR-Egger regression intercept and Mendelian randomization pleiotropy residual sum and outlier were used as sensitivity analyses to detect heterogeneity and multilevel pleiotropy. Additionally, reverse MR was conducted to analyze the causal association between AF and VV. The IVW method indicated a positive causal relationship between VV and AF (odds ratio = 1.1571, 95% confidence interval = 1.0810–1.2384, *P* = 2.59 × 10^−5^). Reverse MR analysis shows no potential reverse causal relationships. The results showed a significant causal effect of VV on AF, suggesting that VV may increase the risk of developing AF. It also elaborates on the common risk factors and pathophysiological conditions between VV and AF.

## 1. Introduction

Varicose veins (VV) is characterized by the loss of function of the venous valves, leading to twisting and dilation of veins. Studies have shown that 70% to 80% of patients with VV have a family history.^[1,2]^ Genetic polymorphisms associated with susceptibility are important in the occurrence and development of VV, and inflammatory cells play a central role in their pathophysiology.^[3]^

Atrial fibrillation (AF) is one of the most common types of arrhythmia. Due to the aging population and the increasing incidence of heart and brain-related diseases, the prevalence of AF is also continuously rising, having doubled in the past 30 years,^[4–6]^ affecting over 33 million people worldwide.^[7]^ Therefore, it is crucial to identify and understand the factors that contribute to the risk of AF.

Although the link between VV and heart disease is unclear, recent research indicates a possible connection between VV and cardiovascular diseases.^[8–10]^ For instance, a cohort study in Taiwan found that AF prevalence was significantly higher in patients with VV than in those without VV.^[11]^ Although some studies have explored the relationship between them, it is important to note that most of these are observational and have issues like uncontrolled confounding factors and reverse causation bias. The exact causal relationship between VV and AF is unclear. However, given the lifetime risk of AF and its high prevalence and mortality rates,^[5,12,13]^ it is necessary to study the causal relationship between VV and AF in order to reduce the disease burden.

In practice, Mendelian randomization (MR) has been widely used to assess the potential causal relationships between various exposures and clinical outcomes. Because allele randomization occurs prior to disease onset, MR analysis can overcome biases introduced by reverse causation compared to traditional observational studies. Therefore, while the MR method is conceptually similar to randomized controlled trials, it is more widely applicable and cost-effective. In addition, random segregation at conception and independent categorization of genetic polymorphisms allow MR analyses to test for causal associations between modifiable exposures and disease using genetic variants (single nucleotide polymorphisms [SNPs]) as instrumental variables (IVs), minimizing the influence of confounding factors.^[14]^ Genome-wide association studies (GWAS) enable us to explore causal relationships.

Thus, we aim to evaluate the causal relationship between VV and AF by using a bidirectional 2-sample Mendelian randomization method. The GWAS summary statistics for VV and AF both come from the FinnGen Consortium R11 released dataset.

## 2. Materials and methods

### 2.1. Research design

**Figure 1.**
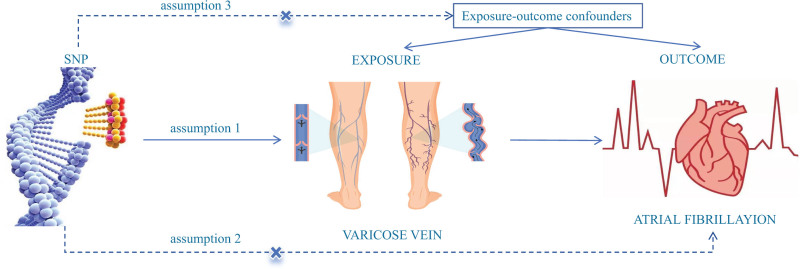
Three basic assumptions of Mendelian randomization.

Following the STROBE-MR guidelines, this study conducts a 2-way bidirectional 2-sample Mendelian randomization analysis.^[15]^ MR is founded on 3 primary assumptions: a strong association exists between IVs and the exposure factor; IVs must be independent of known and unknown confounding factors, ensuring they do not distort the exposure-outcome relationship; exclusion restriction, which states that IVs should only influence the outcome through their connection to the exposure.^[16]^ See Figure 1. This study uses publicly available data, so it does not require informed consent or ethical approval.

### 2.2. Data source

GWAS summary statistics for both VV and AF are sourced from the released dataset of the FinnGen Consortium R11. Launched in 2017, the FinnGen study is a comprehensive national research project that merges genetic data from the Finnish biobank with digital health records from the national registries, providing a wealth of GWAS data that contributes to high-quality exposure and outcome data. This GWAS included a sample of 34,542 cases and 392,860 controls for the VV group, and 55,853 cases and 231,952 controls for the AF group. See Table 1.As this study was a reanalysis of previously collected and published data, no additional ethical approval was required.

**Table 1:** Details of studies included in the Mendelian randomization analyses.

Disease	GWAS ID	Database	Case/control	Population	Source
VV	finngen_R11_I9_VARICVE	Finngen	34,542/392,860	European	https://r11.finngen.fi/
AF	finngen_R11_I9_AF	Finngen	55,853/231,952	European	

AF = atrial fibrillation, GWAS = genome-wide association study, VV = varicose veins.

### 2.3. Selection of IVs

**Figure 2.**
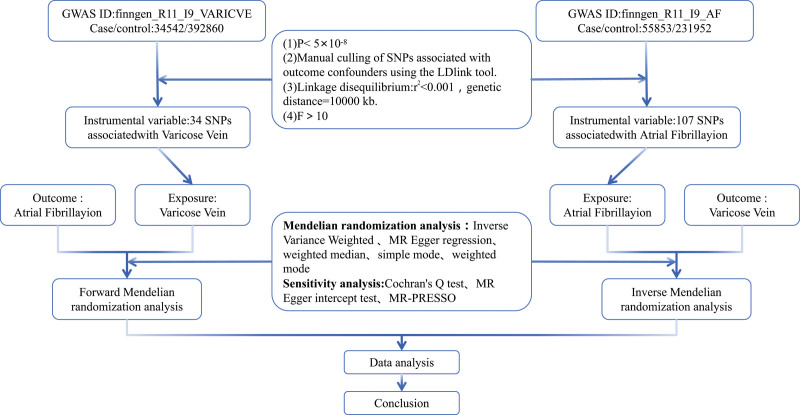
Study design of the current study: Mendelian randomization. GWAS = genome-wide association study, MR = Mendelian randomization, MR-PRESSO = Mendelian randomization pleiotropy residual sum and outlier, SNPs = single nucleotide polymorphisms.

This study selects IVs through strict quality control to ensure the stability and reliability of MR analysis.^[17]^ The selection of SNPs used as IVs is based on the following criteria: SNPs must meet the genome-wide significance threshold (*P* < 5 × 10^−8^) to ensure a strong correlation between IVs and exposure factors; SNPs must be independent of the outcome (excluding confounding conditions),^[18]^ meaning that SNPs only affect the risk of outcome through exposure factors, without other pathways influencing the occurrence of the outcome; they must be independent of any confounding factors related to risk and outcome, meaning they are unrelated to other factors that simultaneously affect both exposure and outcome. This study uses the LDlink tool (https://ldlink.nci.nih.gov/) to search for phenotypes associated with instrumental variables at the genome-wide significance level, manually excluding all SNPs related to confounding factors of AF outcome events from VV^[19]^; similarly, in reverse MR, all SNPs related to confounding factors of VV outcome events in AF are excluded. After selecting IVs, all SNPs undergo linkage disequilibrium processing (defined as *r*^2^ < 0.001 within a 10,000 kbp window based on the 1000 Genomes Project European panel),^[20]^ and ensuring that the *F* statistic of SNPs [*F* statistic=(β/SE)²]>10 to avoid weak instrument bias.^[21]^ Potential pleiotropic SNPs are removed using the Mendelian randomization pleiotropy residual sum and outlier (MR-PRESSO) method. The analysis process is shown in Figure 2.

### 2.4. Data analysis

This study uses the IVW method as the primary analysis method.^[19]^ However, it relies on the assumption that all genetic variations are valid instrumental variables, which may not always be the case.^[22]^ Therefore, we also employed additional methods, including the weighted median, MR-Egger regression, simple mode, and weighted mode, to supplement the results. All MR analyses were carried out using R software, “Two Sample MR” and “MR PRESSO” packages. Additionally, forest plots were generated using the “forestplot” package.

Sensitivity analysis includes heterogeneity testing, horizontal pleiotropy testing, and individual deletion testing. Cochran’s *Q* test is used to assess the heterogeneity of SNPs, and the presence of heterogeneity may lead to increased uncertainty in the analysis results.^[23]^ Through these testing methods, the consistency of the impact of different genetic variations on disease outcomes can be evaluated. If the Cochran’s *Q* test is not statistically significant (*P* > .05), it indicates that the analysis results do not show significant heterogeneity.^[24]^ The MR Egger intercept test is used to detect horizontal pleiotropy, with *P* > .05 indicating no horizontal pleiotropy.^[25]^ MR-PRESSO can provide relatively stable causal effect values in the presence of outliers. It includes testing for horizontal pleiotropy, identifying outliers among instrumental variables, and calculating causal effects after removing outliers, as well as comparing the differences in causal associations before and after correction. Therefore, the global test of MR-PRESSO analysis is used for horizontal pleiotropy testing, with *P* > .05 indicating no horizontal pleiotropy.^[26]^ In the MR-PRESSO outlier test, we manually remove outliers when there are significant outliers.^[27]^ The leave-one-out analysis evaluates the impact of individual SNPs on the results. The risk relationship between VV and AF was expressed as odds ratio and its 95% confidence interval, which indicated a significant causal relationship if *P* < .05.^[28]^

## 3. Results

### 3.1. MR analysis

**Figure 3.**
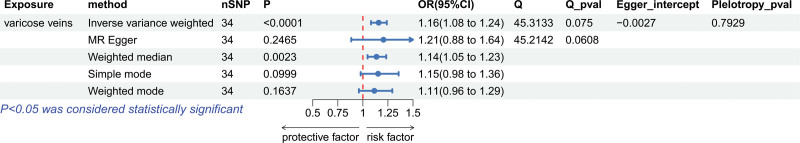
Result of Mendelian randomization in evaluating the casual association between varicose vein and atrial fibrillation. SNP = single nucleotide polymorphism.

The forward MR analysis set VV as the exposure and AF as the outcome. The results indicate a potential positive correlation between VV and AF. After rigorous statistical analysis and data preprocessing, a total of 34 IVs were screened out after excluding SNPs associated with confounding factors (specific information can be found in the Table S1, Supplemental Digital Content, http://links.lww.com/MD/O400) According to the IVW results (see Fig. 3), VV (odds ratio = 1.1571, 95% confidence interval = 1.0810–1.2384, *P* = 2.59 × 10^−5^) is associated with an increased risk of AF. Detailed information can be found in Table S2, Supplemental Digital Content, http://links.lww.com/MD/O400.

### 3.2. Sensitivity and pleiotropy analysis

**Figure 4.**
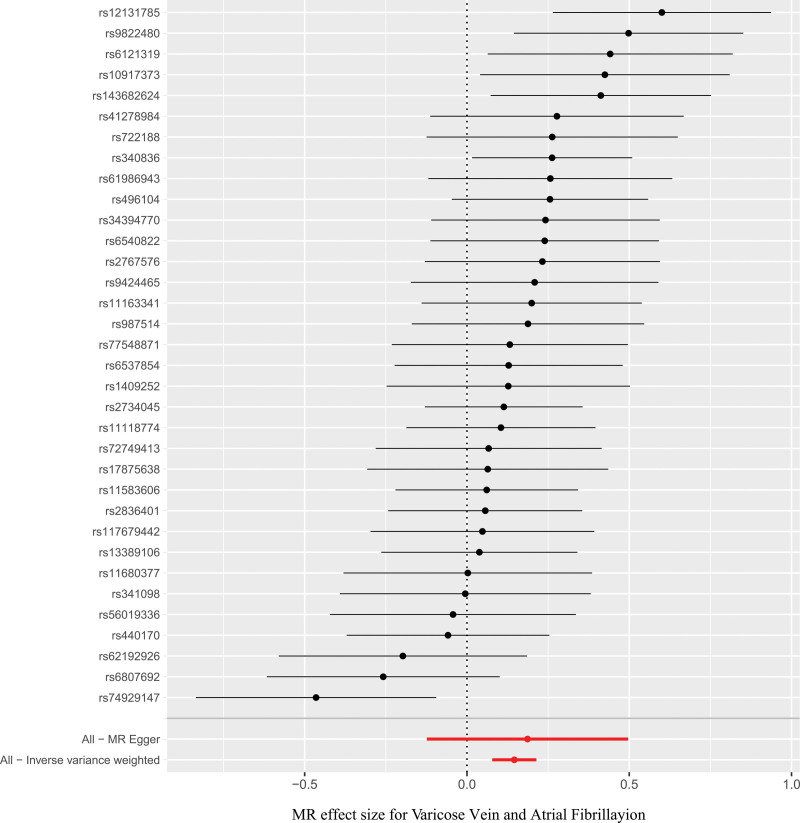
Forest plot of the association between varicose vein and atrial fibrillation. MR = Mendelian randomization.

**Figure 5.**
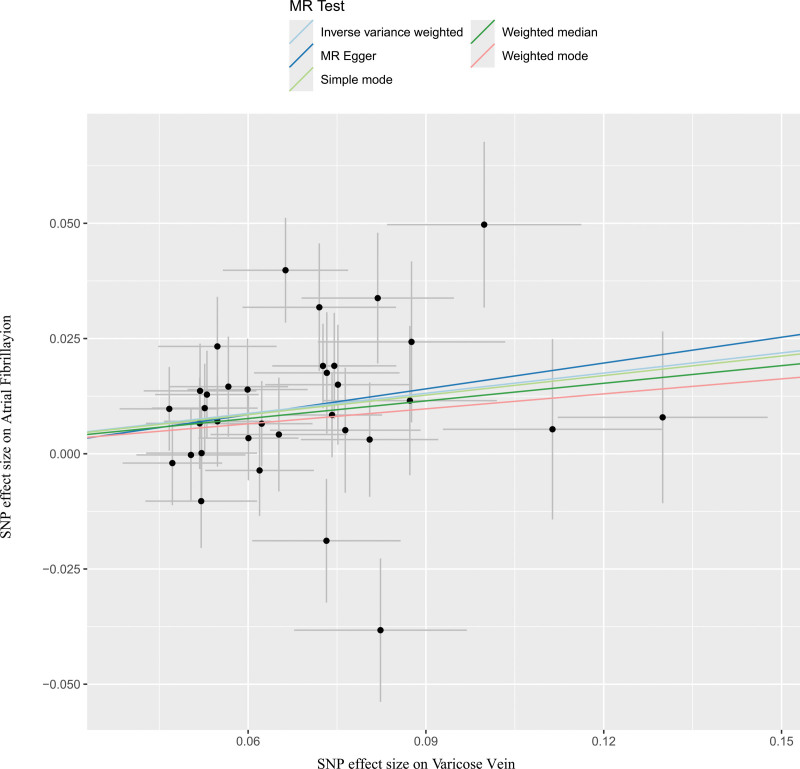
Scatter plots of the association between varicose vein and atrial fibrillation. MR = Mendelian randomization, SNP = single nucleotide polymorphism.

**Figure 6.**
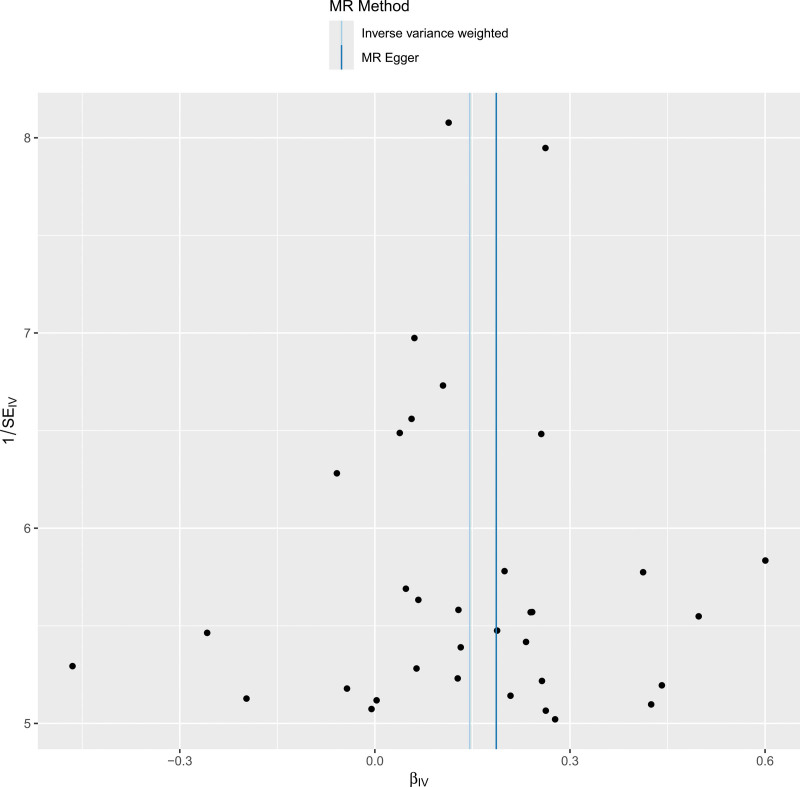
Funnel plots of the association between varicose vein and atrial fibrillation. MR = Mendelian randomization.

**Figure 7.**
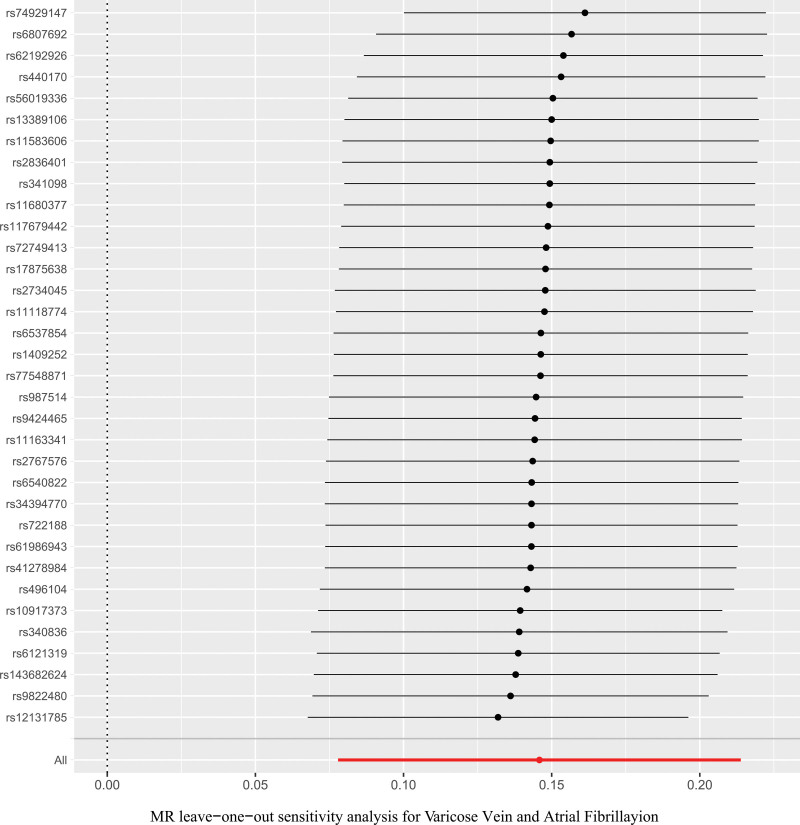
Leave-one-out sensitivity analysis of the relationship between varicose vein and atrial fibrillation. MR = Mendelian randomization.

In a series of sensitivity analyses, Cochran’s *Q* test results showed *P* > .05, indicating no heterogeneity. The MR-Egger regression intercept (*P* = .793) and MR-PRESSO (*P* = .105) suggest that there is no horizontal pleiotropy. Additionally, no significant asymmetry was found in the funnel plot, which also indicates that publication bias and horizontal pleiotropy can be considered negligible. No potential outliers were observed in the scatter plots. The leave-one-out method indicated that removing any single SNP would not significantly affect the overall results, confirming the stability of the causal relationship in this MR analysis. See Figures 4 to 7. All the results of the sensitivity analysis are in Table S3 to S5, Supplemental Digital Content, http://links.lww.com/MD/O400.

### 3.3. Reverse MR analysis

The reverse MR analysis set AF as the exposure and VV as the outcome. After excluding SNPs related to confounding factors, a total of 107 IVs related to VV were screened. The IVW results showed no significant causal relationship between AF and VV (*P* > .05). The results of the reverse MR analysis suggest that there is no significant causal relationship between AF and VV. Please refer to Table S6, Supplemental Digital Content, http://links.lww.com/MD/O400.

## 4. Discussion

Our study conducted a bidirectional, 2-sample MR analysis for VV and AF for the first time, demonstrating a strong causal relationship between VV and the risk of AF in individuals of European ancestry. Furthermore, sensitivity analyses confirmed a positive correlation between the 2 conditions, indicating that the causal effect of VV on AF is independent of confounding factors. However, in the reverse MR analysis, there was insufficient evidence to support a significant causal relationship between AF and the prevalence of VV.

Previously, scholars have suggested that there is a certain correlation between VV and AF.^[29]^ We consider that this may be attributed to the issue of blood stasis inherent in the varicose veins themselves,^[30]^ coupled with the fact that VV patients often experience discomfort from heaviness and swelling in their legs, which results in lower levels of physical activity.^[31]^ This further promotes an increase in blood viscosity, potentially becoming a risk factor for the occurrence of AF. Additionally, there are cases of AF occurring postoperatively in VV patients. For instance, Cho et al^[32]^ reported that a 78-year-old healthy male patient developed sudden upper abdominal pain on the second day after undergoing VV stripping and ligation on his right leg, with new-onset AF observed on the electrocardiogram. Furthermore, VV can lead to reduced venous return and hemodynamic abnormalities, and changes in hemodynamics have many direct effects on the structure and function of the left atrium and left ventricle, suggesting that AF is a consequence of organ damage caused by impaired hemodynamic status.^[33]^ A recent cohort study based on the Korean population found a potential correlation between VV and an increased risk of AF occurrence, proposing that VV should be considered a contributing factor for the development of AF.^[34]^

The pathogenesis of AF involves complex inflammatory responses and electrophysiological changes.^[35,36]^ A clinical trial study found that the levels of interleukin-6 (IL-6) and tumor necrosis factor-alpha (TNF-α) in the blood of VV patients were significantly higher than those in the healthy control group.^[37]^ A case-control study showed that the concentrations of IL-6 and TNF-α in AF patients were independently associated with AF.^[35]^ Furthermore, the role of IL-6 in atrial remodeling and fibrosis has been validated, and inhibiting IL-6 helps improve atrial electrophysiological characteristics and structure, reducing AF susceptibility.^[38]^ TNF-α can increase AF susceptibility by altering the expression and function of ion channels, prolonging the action potential duration of atrial myocytes, and increasing irregularity.^[39,40]^

The study by Kowalska et al^[41]^ pointed out that serum samples obtained from functionally impaired varicose veins had significantly higher levels of vascular cell adhesion molecule-1 (VCAM-1) compared to samples from the elbow vein. VCAM-1, as a key mediator for leukocytes to adhere and migrate from circulation to surrounding tissues, its expression in the endocardium can promote thrombosis and left atrial thrombus formation, suggesting that increased VCAM-1 expression may be an important bridge connecting the inflammatory mechanisms of atrial endocardial thrombus formation with pro-thrombotic mechanisms.^[42–45]^ Therefore, some scholars have suggested using VCAM-1 as a predictive biomarker for atrial fibrillation.^[45]^

Based on the knowledge that von Willebrand factor (vWF) levels in the circulation of VV patients are higher than those in healthy individuals, Kowalska et al^[41]^ further observed that serum from VV patients can induce the upregulation of gene expression for vWF synthesis in senescent cells, reinforcing the enhanced pro-thrombotic characteristics of VV serum on endothelial cells. Conway et al^[46]^ found a positive correlation between AF and vWF in a large community study based on an elderly population. Elevated vWF levels have gradually been recognized as an independent predictor of AF in the general population, and a well-designed meta-analysis indicated that circulating vWF levels in AF patients are significantly higher than those in the sinus rhythm population, further confirming this assertion.^[47,48]^

As an important chemical mediator, monocyte chemoatgulant protein-1 (MCP-1) can attract white blood cells to migrate towards the venous wall, exacerbating the inflammatory response and driving vascular remodeling.^[49]^ Research by Arase et al^[50]^ found that MCP-1 significantly accumulates in the endothelial cells of varicose vein walls, and compared to samples from the elbow vein, the levels of MCP-1 and markers of endothelial dysfunction in samples from varicose veins are significantly upregulated. Notably, the damaged endothelial cells in varicose veins continue to produce inflammatory factors and chemical mediators, leading to an increasing level of these substances in patients as the disease progresses.^[51]^ It has been described that MCP-1 can recruit monocytes to adhere to endothelial cells and differentiate into macrophages, exacerbating the inflammatory response and involving various cardiac metabolic diseases.^[52]^ Li et al^[53]^ reported that MCP-1 levels are independently associated with AF, with no significant differences among different types of AF (paroxysmal, persistent, permanent).

In addition, changes in the extracellular matrix (ECM) components and incomplete functions are considered key factors in the progression of VV.^[54]^ Specifically, matrix metalloproteinases (MMPs) can lead to venous wall dilation by degrading the ECM, thereby accelerating the formation of VV.^[55]^ An experimental study on animals revealed that prolonged increases in venous wall tension can weaken venous contraction ability, and this change is closely related to enhanced MMP activity.^[55]^ At the cardiac tissue level, the ECM not only plays a role in providing structural support for cardiomyocytes, ensuring the structural integrity and geometric stability of the heart, but also interacts dynamically with cardiomyocytes during electrical signal conduction.^[56]^ Given this, some researchers speculate that the occurrence of AF may be associated with changes in the atrial ECM components. By comparing the levels of type I and type III collagen, as well as related MMPs and tissue inhibitors of metalloproteinases in the atria of patients with end-stage heart failure with or without AF, they found a significant correlation between atrial ECM remodeling and the occurrence of AF.^[57]^ Liu et al^[58]^ explicitly pointed out in their comprehensive meta-analysis that the increase in MMP levels in atrial tissue and the decrease in tissue inhibitors of metalloproteinase levels in circulation are significantly statistically correlated with an increased risk of AF. This also provides a basis for better understanding the relationship between VV and AF.

### 4.1. Limitation of study

This study still has several limitations: First, the analysis was conducted only on the European population, which, while helping to eliminate biases arising from exposure and outcome factors not belonging to the same racial background, somewhat limits the generalizability of the conclusions to non-European populations. In light of this, future research should aim to expand its coverage to include groups of different races and ethnicities in order to obtain more comprehensive and universally applicable insights; Second, due to the complexity of disease progression, MR studies can only reduce confounding factors to a certain extent and cannot completely eliminate them, which may introduce potential bias into our results; Third, the potential biological mechanisms between VV and AF have not been sufficiently studied. Fourth, due to the lack of sufficient clinical research literature and detailed clinical information, it is currently not possible to conduct subgroup analyses or meta-analyses to explore potential sources of bias and heterogeneity.

## 5. Conclusion

In summary, this study provides strong evidence for the first time, based on individuals of European descent, that VV is a risk factor for AF from the genetic and causal relationship perspective. This not only enriches our understanding of cardiovascular diseases but also opens new perspectives for innovative clinical management strategies. The study suggests that in clinical practice, physicians need to fully recognize the potential risk of AF in patients with VV, especially among the elderly and those with cardiovascular diseases or other conditions that impair cardiac function. For such patients, it is crucial to pay close attention to their cardiovascular health, closely monitor fluctuations in inflammatory factor levels with atrial fibrillation, and implement dynamic electrocardiogram examinations aimed at the early identification of AF, while comprehensively assessing the risk probability of AF episodes. Furthermore, given the current lack of effective predictive models for varicose vein-related AF, we look forward to future research that can further refine and accurately explore the intrinsic relationship between the severity of varicose veins and atrial fibrillation, to achieve earlier precise intervention and management. Finally, by organizing multi-center, large-sample clinical trials to systematically evaluate the intervention effects of different treatment plans on diseases associated with VV and AF, solid evidence will be provided to formulate more precise and effective clinical management strategies, thereby promoting continuous progress in clinical practice in this field.

## Author contributions

**Conceptualization:** Weiyue Chen, Na Jing, Yannan Wang.

**Funding acquisition:** Weiyue Chen, Yannan Wang.

**Investigation:** Weiyue Chen, Qingzhi Liu, Hong Mao, Yannan Wang.

**Methodology:** Weiyue Chen, Na Jing, Qingzhi Liu.

**Resources:** Na Jing, Qingzhi Liu.

**Supervision:** Qingzhi Liu, Hong Mao, Xiangyu Wang, Boxun Chen, Yannan Wang.

**Writing – original draft:** Weiyue Chen.

**Writing – review & editing:** Weiyue Chen, Yannan Wang.

## Supplementary Material



## References

[1] Mead GE Elder A Flapan AD Cordina J. Electrical cardioversion for atrial fibrillation and flutter. Cochrane Database Syst Rev. 2017;11:CD002903. 29140555 10.1002/14651858.CD002903.pub3 PMC6485992

[2] Merlen JF Coget J Larère J. L’hérédité des varices [Heredity of varices]. Phlebologie. 1967;20:213–6. 6056860

[3] Raffetto JD. Pathophysiology of chronic venous disease and venous ulcers. Surg Clin North Am. 2018;98:337–47. 29502775 10.1016/j.suc.2017.11.002

[4] Go AS Hylek EM Phillips KA. Prevalence of diagnosed atrial fibrillation in adults: national implications for rhythm management and stroke prevention: the AnTicoagulation and Risk Factors in Atrial Fibrillation (ATRIA) Study. JAMA. 2001;285:2370–5. 11343485 10.1001/jama.285.18.2370

[5] Guo Y Tian Y Wang H Si Q Wang Y Lip GYH. Prevalence, incidence, and lifetime risk of atrial fibrillation in China: new insights into the global burden of atrial fibrillation. Chest. 2015;147:109–19. 24921459 10.1378/chest.14-0321

[6] Karamitanha F Ahmadi F Fallahabadi H. Difference between various countries in mortality and incidence rate of the atrial fibrillation based on human development index in worldwide: data from global burden of disease 2010-2019. Curr Probl Cardiol. 2023;48:101438. 36191694 10.1016/j.cpcardiol.2022.101438

[7] Chung MK Refaat M Shen WK; ACC Electrophysiology Section Leadership Council. Atrial fibrillation: JACC council perspectives. J Am Coll Cardiol. 2020;75:1689–713. 32273035 10.1016/j.jacc.2020.02.025

[8] Chang SL Huang YL Lee MC. Association of varicose veins with incident venous thromboembolism and peripheral artery disease. JAMA. 2018;319:807–17. 29486040 10.1001/jama.2018.0246 PMC5838574

[9] Wu NC Chen ZC Feng IJ. Severe varicose veins and the risk of mortality: a nationwide population-based cohort study. BMJ Open. 2020;10:e034245. 10.1136/bmjopen-2019-034245 PMC7311034 32565451

[10] Mäkivaara LA Ahti TM Luukkaala T Hakama M Laurikka JO. Persons with varicose veins have a high subsequent incidence of arterial disease: a population-based study in Tampere, Finland. Angiology. 2007;58:704–9. 18216380 10.1177/0003319707299202

[11] Hu WS Lin CL. Association between varicose vein and atrial fibrillation-a population-based study in Taiwan. Phlebology. 2022;37:535–9. 35466790 10.1177/02683555221095299

[12] Shi S Tang Y Zhao Q; China Atrial Fibrillation Center Project Team. Prevalence and risk of atrial fibrillation in China: a national cross-sectional epidemiological study. Lancet Reg Health West Pac. 2022;23:100439. 35800039 10.1016/j.lanwpc.2022.100439 PMC9252928

[13] Lippi G Sanchis-Gomar F Cervellin G. Global epidemiology of atrial fibrillation: an increasing epidemic and public health challenge. Int J Stroke. 2021;16:217–21. 31955707 10.1177/1747493019897870

[14] Lawlor DA Harbord RM Sterne JA Timpson N Davey SG. Mendelian randomization: using genes as instruments for making causal inferences in epidemiology. Stat Med. 2008;27:1133–63. 17886233 10.1002/sim.3034

[15] Skrivankova VW Richmond RC Woolf BAR. Strengthening the reporting of observational studies in epidemiology using mendelian randomisation (STROBE-MR): explanation and elaboration. BMJ. 2021;375:n2233. 34702754 10.1136/bmj.n2233 PMC8546498

[16] Emdin CA Khera AV Kathiresan S. Mendelian randomization. JAMA. 2017;318:1925–6. 29164242 10.1001/jama.2017.17219

[17] Zhang B He R Yao Z. Exploring causal relationships between circulating inflammatory proteins and thromboangiitis obliterans: a Mendelian randomization study. Thromb Haemost. 2024;124:1075–83. 38788766 10.1055/s-0044-1786809 PMC11518616

[18] Burgess S Small DS Thompson SG. A review of instrumental variable estimators for Mendelian randomization. Stat Methods Med Res. 2017;26:2333–55. 26282889 10.1177/0962280215597579 PMC5642006

[19] Li YX Barton JP. Estimating linkage disequilibrium and selection from allele frequency trajectories. Genetics. 2023;223:iyac189. 36610715 10.1093/genetics/iyac189 PMC9991507

[20] Purcell S Neale B Todd-Brown K. PLINK: a tool set for whole-genome association and population-based linkage analyses. Am J Hum Genet. 2007;81:559–75. 17701901 10.1086/519795 PMC1950838

[21] Palmer TM Lawlor DA Harbord RM. Using multiple genetic variants as instrumental variables for modifiable risk factors. Stat Methods Med Res. 2012;21:223–42. 21216802 10.1177/0962280210394459 PMC3917707

[22] Xiang M Wang Y Gao Z. Exploring causal correlations between inflammatory cytokines and systemic lupus erythematosus: a Mendelian randomization. Front Immunol. 2023;13:985729. 36741410 10.3389/fimmu.2022.985729 PMC9893779

[23] Sekula P Del Greco M F Pattaro C Köttgen A. Mendelian randomization as an approach to assess causality using observational data. J Am Soc Nephrol. 2016;27:3253–65. 27486138 10.1681/ASN.2016010098 PMC5084898

[24] Burgess S Butterworth A Thompson SG. Mendelian randomization analysis with multiple genetic variants using summarized data. Genet Epidemiol. 2013;37:658–65. 24114802 10.1002/gepi.21758 PMC4377079

[25] Bowden J Davey Smith G Burgess S. Mendelian randomization with invalid instruments: effect estimation and bias detection through Egger regression. Int J Epidemiol. 2015;44:512–25. 26050253 10.1093/ije/dyv080 PMC4469799

[26] Zhou X Wang L Xiao J. Alcohol consumption, DNA methylation and colorectal cancer risk: results from pooled cohort studies and Mendelian randomization analysis. Int J Cancer. 2022;151:83–94. 35102554 10.1002/ijc.33945 PMC9487984

[27] Verbanck M Chen CY Neale B Do R. Detection of widespread horizontal pleiotropy in causal relationships inferred from Mendelian randomization between complex traits and diseases. Nat Genet. 2018;50:693–8. 29686387 10.1038/s41588-018-0099-7 PMC6083837

[28] Chen W Zhang T Zhang H. Causal relationship between type 2 diabetes and glioblastoma: bidirectional Mendelian randomization analysis. Sci Rep. 2024;14:16544. 39020091 10.1038/s41598-024-67341-x PMC11255221

[29] Yetkin E Atmaca H Yalta K. Atrial fibrillation and peripheral varicose vein: where is the connection? Phlebology. 2023;38:133–4. 36592347 10.1177/02683555221148835

[30] Oklu R Habito R Mayr M. Pathogenesis of varicose veins. J Vasc Interv Radiol. 2012;23:33–9; quiz 40. 22030459 10.1016/j.jvir.2011.09.010

[31] Raetz J Wilson M Collins K. Varicose veins: diagnosis and treatment. Am Fam Physician. 2019;99:682–8. 31150188

[32] Cho J Lee D. Postoperative new-onset atrial fibrillation causing acute embolic occlusion of the superior mesenteric artery: a case report. Medicine (Baltim). 2021;100:e25700. 10.1097/MD.0000000000025700 PMC8084075 33907150

[33] Wachtell K. Atrial fibrillation is target organ damage caused by an impaired haemodynamic state. Heart. 2018;104:1234–5. 29574412 10.1136/heartjnl-2017-312778

[34] Choi S Leem GH Song TJ. Association of varicose veins with incidence risk of atrial fibrillation: a population-based cohort study. Int J Surg. 2024;110:5704–12. 39166948 10.1097/JS9.0000000000002036 PMC11392101

[35] Bai W Liu ZQ He PY Muhuyati. The role of IL-6, IL-10, TNF-α and PD-1 expression on CD4 T cells in atrial fibrillation. Heliyon. 2023;9:e18818. 37636377 10.1016/j.heliyon.2023.e18818 PMC10448416

[36] Su KN Ma Y Cacheux M. Atrial AMP-activated protein kinase is critical for prevention of dysregulation of electrical excitability and atrial fibrillation. JCI Insight. 2022;7:e141213. 35451373 10.1172/jci.insight.141213 PMC9089788

[37] Signorelli SS Malaponte MG Di Pino L Costa MP Pennisi G Mazzarino MC. Venous stasis causes release of interleukin 1beta (IL-1beta), interleukin 6 (IL-6) and tumor necrosis factor alpha (TNFalpha) by monocyte-macrophage. Clin Hemorheol Microcirc. 2000;22:311–6. 11081468

[38] Li X Wu X Chen X. Selective blockade of interleukin 6 trans-signaling depresses atrial fibrillation. Heart Rhythm. 2023;20:1759–70. 37633428 10.1016/j.hrthm.2023.08.026

[39] Kao YH Chen YC Cheng CC Lee T-I Chen Y-J Chen S-A. Tumor necrosis factor-alpha decreases sarcoplasmic reticulum Ca2^+^-ATPase expressions via the promoter methylation in cardiomyocytes. Crit Care Med. 2010;38:217–22. 19730253 10.1097/CCM.0b013e3181b4a854

[40] Sawaya SE Rajawat YS Rami TG. Downregulation of connexin40 and increased prevalence of atrial arrhythmias in transgenic mice with cardiac-restricted overexpression of tumor necrosis factor. Am J Physiol Heart Circ Physiol. 2007;292:H1561–1567. 17122196 10.1152/ajpheart.00285.2006

[41] Kowalska K Zabel M Wysocka T Khalil RA Raffetto JD Urbanek T. Changes of the serum properties and its effect on the endothelial cells restoration in patients with chronic venous disease treated with sulodexide. J Vasc Surg Venous Lymphat Disord. 2024;12:101941. 38945361 10.1016/j.jvsv.2024.101941 PMC11523325

[42] Goette A Bukowska A Lendeckel U. Angiotensin II receptor blockade reduces tachycardia-induced atrial adhesion molecule expression. Circulation. 2008;117:732–42. 18227384 10.1161/CIRCULATIONAHA.107.730101

[43] Hammwöhner M Ittenson A Dierkes J. Platelet expression of CD40/CD40 ligand and its relation to inflammatory markers and adhesion molecules in patients with atrial fibrillation. Exp Biol Med (Maywood). 2007;232:581–9. 17392495

[44] Breitenstein A Glanzmann M Falk V. Increased prothrombotic profile in the left atrial appendage of atrial fibrillation patients. Int J Cardiol. 2015;185:250–5. 25814212 10.1016/j.ijcard.2015.03.092

[45] Troncoso MF Ortiz-Quintero J Garrido-Moreno V. VCAM-1 as a predictor biomarker in cardiovascular disease. Biochim Biophys Acta Mol Basis Dis. 2021;1867:166170. 34000374 10.1016/j.bbadis.2021.166170

[46] Conway DS Heeringa J Van Der Kuip DA. Atrial fibrillation and the prothrombotic state in the elderly: the Rotterdam Study. Stroke. 2003;34:413–7. 12574552 10.1161/01.str.0000051728.85133.32

[47] Alonso A Tang W Agarwal SK Soliman EZ Chamberlain AM Folsom AR. Hemostatic markers are associated with the risk and prognosis of atrial fibrillation: the ARIC study. Int J Cardiol. 2012;155:217–22. 20965585 10.1016/j.ijcard.2010.09.051 PMC3025309

[48] Weymann A Sabashnikov A Ali-Hasan-Al-Saegh S. Predictive role of coagulation, fibrinolytic, and endothelial markers in patients with atrial fibrillation, stroke, and thromboembolism: a meta-analysis, meta-regression, and systematic review. Med Sci Monit Basic Res. 2017;23:97–140. 28360407 10.12659/MSMBR.902558 PMC5452871

[49] Zolotukhin I Golovanova O Efremova O Golovina V Seliverstov E. Monocyte chemoattractant protein 1 plasma concentration in blood from varicose veins decreases under venoactive drug treatment. Int Angiol. 2022;41:457–63. 36326144 10.23736/S0392-9590.22.04940-9

[50] Arase H Sugasawa N Kawatani Y. Appropriate surgical treatment of symptomatic primary varicose veins decreases systemic inflammatory biomarkers. Ann Vasc Dis. 2019;12:367–71. 31636748 10.3400/avd.oa.19-00011 PMC6766771

[51] Lohr JM Bush RL. Venous disease in women: epidemiology, manifestations, and treatment. J Vasc Surg. 2013;57(4 Suppl):37S–45S. 23522716 10.1016/j.jvs.2012.10.121

[52] Ren W Huang Y Meng S. Salidroside treatment decreases the susceptibility of atrial fibrillation in diabetic mice by reducing mTOR-STAT3-MCP-1 signaling and atrial inflammation. Int Immunopharmacol. 2024;142:113196. 39306893 10.1016/j.intimp.2024.113196

[53] Li J Solus J Chen Q. Role of inflammation and oxidative stress in atrial fibrillation. Heart Rhythm. 2010;7:438–44. 20153266 10.1016/j.hrthm.2009.12.009 PMC2843774

[54] Chen T Liu P Zhang C. Pathophysiology and genetic associations of varicose veins: a narrative review [published online ahead of print January 16, 2024]. Angiology. doi: 10.1177/00033197241227598. 10.1177/00033197241227598 38226614

[55] Raffetto JD Qiao X Koledova VV Khalil RA. Prolonged increases in vein wall tension increase matrix metalloproteinases and decrease constriction in rat vena cava: Potential implications in varicose veins. J Vasc Surg. 2008;48:447–56. 18502086 10.1016/j.jvs.2008.03.004 PMC2575039

[56] Sackner-Bernstein JD. The myocardial matrix and the development and progression of ventricular remodeling. Curr Cardiol Rep. 2000;2:112–9. 10980881 10.1007/s11886-000-0007-4

[57] Xu J Cui G Esmailian F. Atrial extracellular matrix remodeling and the maintenance of atrial fibrillation. Circulation. 2004;109:363–8. 14732752 10.1161/01.CIR.0000109495.02213.52

[58] Liu Y Xu B Wu N. Association of MMPs and TIMPs with the occurrence of atrial fibrillation: a systematic review and meta-analysis. Can J Cardiol. 2016;32:803–13. 26907578 10.1016/j.cjca.2015.08.001

